# An Analysis of Ophthalmic Cases

**Published:** 1920-08-28

**Authors:** 


### An Analysis of Ophthalmic Cases.

The above publication of the Medical Research Council, "An Analysis of 15,584 Ophthalmic Cases treated at a Home Hospital," deals with work carried out at the 2nd London General Hospital, St. Mark's College, Chelsea, 1914-19. In close relation to the work the Medical Research Council have undertaken for the Army Council in the compilation of the formal Medical Statistics of the War, they have provided accessory help in various directions for the collection and preservation of medical records and of the after-histories of military patients, with a view both to the present guidance of medical officers and to the future purposes of the Medical History of the War. Copies may be obtained from all booksellers or from H.M. Stationery Office. (Medical Research Council. Statistical Report No. 6. Price 6d., post free 7d.)

